# The advance care planning PREPARE study among older Veterans with serious and chronic illness: study protocol for a randomized controlled trial

**DOI:** 10.1186/s13063-015-1055-9

**Published:** 2015-12-12

**Authors:** Rebecca Sudore, Gem M. Le, Ryan McMahon, Mariko Feuz, Mary Katen, Deborah E. Barnes

**Affiliations:** Division of Geriatrics, Department of Medicine, University of California, San Francisco, 3333 California St. Suite 380, San Francisco, CA 94143 USA; San Francisco Veterans Administration Medical Center, 4150 Clement Street, #151R, San Francisco, CA 94121 USA; Division of General Internal Medicine, San Francisco General Hospital, Center for Vulnerable Populations, University of California, San Francisco, 2789 25th Street Suite 350, San Francisco, CA 94110 USA; Department of Psychiatry, University of California, San Francisco, CA USA; Department of Epidemiology & Biostatistics, University of California, San Francisco, CA USA

**Keywords:** advance care planning, aging, medical decision-making, randomized trial, chronic disease management, geriatrics, veterans, health behavior, advance directive, vulnerable populations

## Abstract

**Background:**

Advance care planning (ACP) is a process whereby patients prepare for medical decision-making. The traditional objective of ACP has focused on the completion of advance directives. We have developed a new paradigm of ACP focused on preparing patients and their loved ones for communication and informed medical decision-making. To operationalize this new paradigm of ACP, we created an interactive, patient-centered website called PREPARE (www.prepareforyourcare.org) designed for diverse older adults.

**Methods/Design:**

This randomized controlled trial with blinded outcome assessment is designed to determine the efficacy of PREPARE to engage older Veterans in the ACP process. Veterans who are ≥ 60 years of age, have ≥ two medical conditions, and have seen a primary care physician ≥ two times in the last year are being randomized to one of two study arms. The PREPARE study arm reviews the PREPARE website and an easy-to-read advance directive. The control arm only reviews the advance directive. The primary outcome is documentation of an advance directive and ACP discussions. Other clinically important outcomes using validated surveys include ACP behavior change process measures (knowledge, contemplation, self-efficacy, and readiness) and a full range of ACP action measures (identifying a surrogate, identifying values and goals, choosing leeway or flexibility for the surrogate, communicating with clinicians and surrogates, and documenting one’s wishes). We will also assess satisfaction with decision-making and Veteran activation within primary care visits by direct audio recording. To examine the outcomes at 1 week, 3 months, and 6 months between the two study arms, we will use mixed effects linear, Poisson, or negative binomial regression and mixed effects logistic regression.

**Discussion:**

This study will determine whether PREPARE increases advance directive completion rates and engagement with the ACP process. If PREPARE is efficacious, it could prove to be an easy and effective intervention to help older adults engage in the ACP process within or outside of the medical environment. PREPARE may also help older adults communicate their medical wishes and goals to their loved ones and clinicians, improve medical decision-making, and ensure their wishes are honored over the life course.

**Trial registration:**

ClinicalTrials.gov NCT01550731. Registered on 8 December 2011.

## Background

More than 10 million Veterans who are over age 65 will face complex decisions over the course of chronic illness [1], yet most are unprepared to do so [2, 3]. Inadequate preparation leads to uninformed choices, lack of empowerment in clinical encounters, and added stress for Veterans, their families and surrogate decision makers [4–8]. Interventions to prepare Veterans for complex decisions and to communicate their wishes and goals for medical care to surrogates and clinicians are lacking.

Advance care planning is a process whereby people identify and communicate their goals for future medical care. In the old, narrow paradigm of advance care planning (ACP), the objective was focused on having patients make decisions about life-prolonging procedures, such as cardiac resuscitation, and to document these choices in an advance directive. However, Veterans are making many decisions over the course of their lives, and many Veterans and their loved ones are unprepared to make these decisions or to understand and complete legal advance directive forms, which are often used for advance care planning. More preparation is needed to achieve comprehensive ACP, and ACP guides must meet the literacy needs of older adults and Veterans (5^th^-grade level), as well as address patients’ diverse cultural needs [9–12].

Our prior work addressed some of these barriers to meaningful ACP for older adults. We designed and tested an advance directive written at a 5^th^-grade reading level among 205 chronically ill, diverse, older adults at a county hospital in San Francisco. The easy-to-read directive was preferred over a standard directive, especially among those with limited health literacy. It also resulted in significantly greater 6-month advance directive completion rates, doubling the rates from baseline [12]. This easy-to-read advance directive is currently being disseminated in several languages in California [13].

Although the easy-to-read advance directive was shown to be an improvement over standard forms, through additional formative work, we found that most patients go through a series of ACP behavioral steps that extend beyond simply filling out an advance directive. For example, 6 months after exposure to the easy-to-read advance directive, only 13 % completed an advance directive whereas 61 % of older adults contemplated ACP, 56 % discussed ACP with family or friends and 22 % discussed ACP with clinicians [14]. These data support the need to engage patients in a full range of ACP activities in addition to having them complete legal advance directive forms.

Based on our prior work [15] and the work of others [16, 17], we developed and published an updated paradigm of comprehensive ACP that shifts the focus from completion of advance directives to preparing patients to communicate their goals of care with surrogates and to actively participate with clinicians in making the best possible in-the-moment decisions [11]. To operationalize this new paradigm, we created an easy-to-read, culturally-appropriate website called PREPARE (www.prepareforyourcare.org). PREPARE is targeted to a 5^th^- grade reading level and was designed to be completed outside of a clinical setting without supervision or facilitation. PREPARE includes communication training on how to 1) choose and ask a surrogate; 2) clarify and communicate one’s values; 3) discuss leeway in surrogate’s decisions (allow surrogates to use their best judgment); 4) inform clinicians, family, and friends of one’s decisions; and 5) ask clinicians appropriate questions to make informed medical choices [11]. Extensive videos are used to demonstrate, through modeling, how to make ACP decisions and to communicate with surrogates and clinicians [15, 18–20]. Our pilot findings showed that, among diverse older adults, PREPARE significantly improved engagement and behavior change in ACP. Furthermore, it was rated a 9 out of 10 for ease of use, despite limited health literacy and lack of computer experience in this cohort of older adults [15].

The goal of this randomized controlled trial is to determine the efficacy of PREPARE to engage Veterans in the full comprehensive process of ACP. Consistent with prior studies in the field, our primary outcome is advance directive completion. In addition, important secondary outcomes include identifying goals for medical care, communicating with surrogates and clinicians, and making informed medical decisions.

## Methods/Design

### Theoretical foundation

The conceptual framework for the PREPARE intervention and this study has been previously published [15]. In brief, this work rests on the foundation of Social Cognitive Theory, the Interpersonal Communication Competence Model, and Behavior Change Theory [18, 19, 21]. ACP is a complex behavior that often involves people undergoing a series of behavior change steps, which are influenced by knowledge, self-efficacy and readiness. Behavior change is highly influenced by social norms and modeling of these behaviors by others. This framework demonstrates that PREPARE was designed to address important moderator variables such as culture, religion, and literacy. PREPARE incorporates important aspects from the aforementioned theories by including training in communication, goal setting exercises and videos that demonstrate and model ACP behaviors. PREPARE is designed to address modifiable mediators such as knowledge, self-efficacy, and readiness, which then allows individuals to progress through the behavior change stages from pre-contemplation to contemplation and then to readiness and action for the multiple behaviors that compose the process of ACP. Based on the conceptual framework, we developed and validated the ACP Engagement Survey to measure the full construct and process of ACP [22]. Behavioral process measures of behavior change include knowledge, contemplation, self-efficacy, and readiness, whereas ACP actions include identification of a surrogate, identification of values and goals, choosing the level of leeway or flexibility in decision-making for the surrogate, communicating this information with clinicians and surrogate decision makers, and documenting one’s wishes.

### Aims and primary hypotheses

The primary hypothesis of this study is that older Veterans randomized to the PREPARE arm (the PREPARE website plus the easy-to-read advance directive), compared to the control arm (the easy-to-read advance directive alone), will have increased rates of ACP documentation in their medical record (advance directives, clinicians notes, code status orders). Because this is the standard outcome measure for advance care planning, it is the outcome on which we calculated our sample size (see Sample Size Section). However, ACP consists of several behavioral processes (knowledge, contemplation, self-efficacy, and readiness) for several discrete ACP behaviors, including identifying surrogates, identifying values, and communicating this information with clinicians, surrogates, and other loved ones. Therefore, we feel our secondary outcomes and hypotheses are just as clinically meaningful. The secondary hypotheses of this study are that older Veterans randomized to the PREPARE arm, compared to the control arm, will have greater engagement in ACP including behavior change process measures and additional ACP actions beyond advance directive documentation as measured by a validated survey [22]. Secondary hypotheses also include greater Veteran activation and clinician responsiveness within the clinical encounter as measured by audio-recording a subset of clinical visits, Veteran satisfaction with clinician communication, Veteran engagement and satisfaction with decision-making, surrogate-reports of Veteran engagement in ACP behaviors, and fewer reported barriers to ACP for the PREPARE versus control arm. Other outcomes include comparisons of acceptability and usability of the PREPARE website plus an easy-to-read advance directive versus an advance directive alone (Table 2).

### Setting

The study is a randomized controlled trial with blinded outcome ascertainment conducted at the San Francisco Veterans Administration Medical Center (SFVAMC).

### Overview of study procedures

**Fig. 1:**
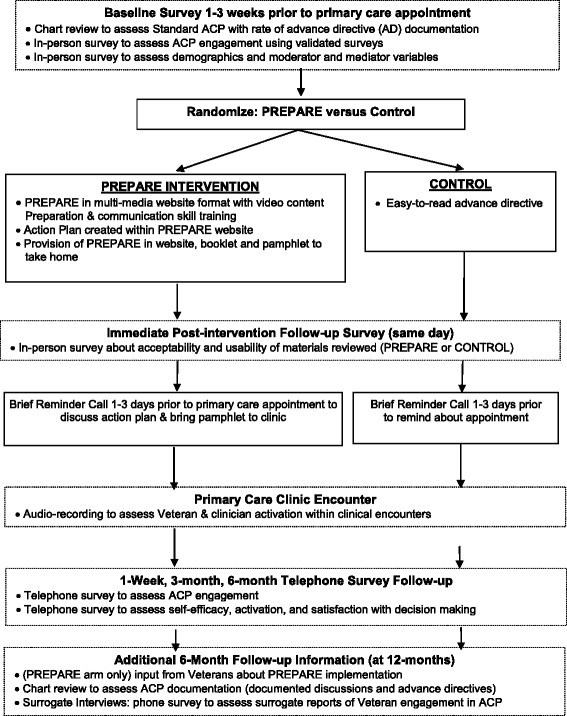
Flowchart of the PREPARE Trial

Veteran recruitment occurs in waves to target upcoming primary care clinic appointments and to allow time for recruitment and scheduling of a baseline interview (Fig. 1, Study Flowchart). Veterans are then randomized to the PREPARE arm (PREPARE website [15], action plan creation within the website, and provision of PREPARE logins and PREPARE materials in pamphlet, booklet and DVD formats to take home) plus an easy-to-read advance directive [12], or the control arm which only receives the easy-to-read advance directive. Outcomes pertaining to ACP engagement behavior are assessed at 1 week, 3 months, and 6 months between the two study arms (Table 2).

### Participants

Table 1 includes a list of all inclusion and exclusion criteria. Briefly, Veterans are eligible for inclusion in this study if they are 60 years of age and older and have two or more chronic or severe medical conditions as determined by any of the ICD-9 codes listed in the Charlson or Elixhauser measures of comorbidity [23, 24]. The Charlson comorbidity index uses ICD-9 codes associated with 22 comorbid conditions such as heart disease and cancer, and the Elixhauser uses 30 acute and chronic conditions associated with in-patient mortality. We use the inclusion criteria of two or more chronic or severe medical conditions to identify a cohort of patients who have medical conditions that may require medical decision-making. Patients also have to have been seen by a primary care clinician at the SFVAMC two or more times in the past year (a marker for established primary care) and have two or more additional outpatient or inpatient visits in the past year (a marker of disease severity and frequent access to care). Patients are excluded if they have dementia; are blind, deaf, or psychotic as determined by their clinician, chart review or study staff; do not have a phone for study contact and follow-up interviews; or do not live within 30 miles of the SFVAMC, which would make an in-person baseline interview difficult (Table 1). We chose to focus our study population on older adults with chronic illness, as our preliminary work suggests that patients may be less likely to engage in ACP if they perceive themselves as “too healthy” [25]. In addition, we chose not to exclude patients with terminal or serious illness because our goal is to move ACP upstream in patients’ disease trajectories and to assess the efficacy of PREPARE across a range of health statuses.

**Table 1:** Inclusion and exclusion criteria by type of study participant

Veteran patient	
Inclusion criteria	60 years of age or older
	Obtains care in the primary care clinics (General Medicine, Geriatrics, and Women’s Clinic) at the San Francisco VA Medical Center
	Has been seen at least twice in the last year by a primary care provider (a measure of established primary care) and had at least two additional visits to the VA in the past year (a measure of frequent the medical center)
Exclusion criteria	Dementia by ICD-9 codes, clinician assessment, chart review or self-report
	Blindness or poor vision by ICD-9 codes, clinician assessment, chart review, self-report of blindness or the inability to read print on a newspaper, or research staff assessment of less than 20/200 vision on the Snellen eye chart with corrective lenses [42].
	Deafness by ICD-9 codes, clinician assessment, self-report, chart review or research staff assessment
	Cognitive impairment as assessed by research staff of any deficits on the validated cognitive assessments Short Portable Mental Status Questionnaire (SPMSQ) [43] and the mini-Cog [44]
	Delirium or psychosis as assessed by a clinician or research staff
	Does not report fluency in English
	No phone for additional study contacts and follow-up interviews
	Active drug or alcohol abuse within the past 3 months determined by clinician assessment, self-report, chart review or research staff assessment
	Patients who report they will be out of town during their scheduled follow-up interview dates outside of a window of 2 months
	Patients who cannot answer consent teach-back questions after three attempts
Surrogate participant	
Inclusion criteria	18 years of age or older
	An enrolled patient must identify the surrogate as someone who could make medical decisions for him or her if needed
	An enrolled patient must give the surrogate’s contact information and give permission to contact their potential surrogate
Exclusion criteria	Self-reported dementia, blindness, or deafness
	Cognitive impairment as assessed by research staff of any deficits on the validated cognitive assessments Short Portable Mental Status Questionnaire (SPMSQ) [43] and the mini-Cog [44]
	Delirium or psychosis as assessed by research staff
	Does not report fluency in English
	No phone for follow-up interviews
	Surrogates who report they will be out of town during their scheduled follow-up interview dates outside of a window of 2 months
	Surrogate for whom we cannot schedule an interview greater than 6 months from the Veteran’s final 6-month follow-up interview date
	Surrogates for whom we have attempted to contact 5 times or more without a response
	Surrogates who cannot answer consent teach-back questions after three attempts

The SFVAMC Veteran population consists of  approximately 4 % women [1]. Therefore, we increased recruitment of women by working closely with the SFVAMC Women’s Clinic and oversampling women through our patient recruitment call lists. To ensure a diverse sample, we also oversampled minorities in an attempt to obtain a cohort of 50 % white and 50 % non-white Veterans. Oversampling is feasible as the racial/ethnic variation of SFVAMC outpatients is approximately 55 % white, 29 % black, 8 % Latino, 6 % Asian/Pacific Islander, and 2 % other [26].

For a subset of enrolled participants, we also include Veteran’s self-identified surrogate decision-makers. Surrogates are included if the Veteran identifies her or him and gives the study team permission to contact them. Surrogates are excluded if they had dementia or are blind, deaf, or psychotic as determined by the study staff (Table 1).

### Recruitment

#### Health Insurance Portability and Accountability Act (HIPAA) waiver

To facilitate recruitment, we obtained a HIPAA waiver to access data from VA administrative records on patients’ names, age, race/ethnicity, gender, phone numbers, addresses, medical record numbers, ICD-9 codes, dates of outpatient primary care clinic appointments in the past year and up to 3 months in the future, hospitalizations and emergency room visits in the past year, and the name of their outpatient primary care provider. All data is stored on secure, password-protected servers or in locked research offices. Access is restricted to the study team for recruitment purposes only.

#### Data extraction

After obtaining a HIPAA waiver, we obtained a list of potential Veteran participants who met initial eligibility criteria described in Table 1 and a list of their primary care physicians.

#### Clinician involvement

Upon completion of the administrative data pulls and identification of potentially eligible Veterans, primary care providers are then sent an e-mail letter informing them about the research study. The email also asks providers to give their permission for our study staff to contact their patients and tell them more about the study. A list of the clinician’s patients is provided, and clinicians are given the option to opt out for all patients, approve for all of their patients, or review the patient list to identify individual patients whom they feel are either appropriate or inappropriate for our study. We also ask permission of clinicians to allow us to send their potentially eligible patients a recruitment letter stating that the clinician gave permission to tell them more about a research study. Clinicians can opt out of having their name used in the patient recruitment letter. Clinicians are informed that if they did not respond to our emails or phone calls within three attempts, we will assume assent for the study. If clinicians do not respond after three attempts over 3 weeks to our requests to grant permission to contact their patients, we send a generic, nonpersonalized recruitment letter describing the study to potential participants on behalf of the study team. If a clinician gives us explicit permission to contact their patients, we inform patients that their individual doctor gave us permission to contact them and tell them about the study. Otherwise, we describe the study without mentioning their clinician.

#### Targeted patient recruitment by letter

The recruitment letter to patients describes the research study and gives patients a toll-free telephone number to call to opt-out or to hear more about the study. Potential participants who do not call study staff to decline participation within 1 week of the mailings are deemed eligible to be contacted by phone to further describe the study and assess willingness to participate and study eligibility. Additional waves of patient recruitment letters will be sent out two to three times per year to enhance our recruitment efforts.

#### Targeted patient recruitment by phone

We attempt to schedule Veterans for the baseline interview and exposure to the intervention 1 to 3 weeks prior to their next upcoming primary care appointment. This is done to standardize the timing between intervention exposure and primary care follow-up. Therefore, for Veterans who do not send back an opt-out letter or call to refuse participation, we attempt recruitment by phone. Targeted data pulls are created to determine which Veterans have upcoming primary care appointments at least 2 weeks in the future. This time frame was selected to allow time to screen and schedule consent and baseline interviews 1 to 3 weeks prior to the primary care visit. The order of Veterans’ names on the recruitment call lists is randomly “scrambled” to prevent biased sampling. In addition, women and minority Veterans are prioritized on the call list in an attempt to oversample during phone recruitment.

#### Recruitment fliers

Study-related fliers written at a 5^th^-grade reading level are posted in approved areas in the primary care clinics at the SFVAMC. Potential participants who read a study flier can opt-in to the study by contacting study staff via our toll-free number.

#### Surrogate recruitment

Once Veterans are enrolled in the study, we obtain a consecutive sample of English-speaking surrogates through Veteran referral. For Veterans who grant us permission to contact their potential surrogate, we obtain contact information for the surrogate from the enrolled Veteran. We also ask the Veteran participant to give the potential surrogate participant our study flier and/or let them know we would be contacting them. Depending upon the type of contact information we are given by the Veteran, we may contact the potential surrogate participant in the clinic, if they accompanied a Veteran participant, or by phone, email, or mail.

### Screening for eligibility of Veterans and surrogates

Interested Veteran participants and surrogates are screened for eligibility based on the exclusion criteria in Table 1. Specifically, research staff exclude individuals who report not having a phone, Veterans who are less than 60 years of age, and Veterans or surrogates who test positive for moderate-to-severe cognitive impairment on the Short Portable Mental Status Questionnaire (SPMSQ) as indicated by more than three errors and who then also fail to recall at least two out of three items on the three-item recall of Mini-Cog [27, 28]. Participants are also excluded if they self-reported having dementia, poor vision (unable to see the words on a newspaper), or being unable to speak English well or very well.

### Consent procedures

#### Written consent for patients

Informed consent will be or has been obtained from all study participants. We use a modified consent process we designed to help vulnerable populations make informed decisions about study participation [29]. This process involves using a consent form written at a 5^th^-grade reading level, reading and paraphrasing the consent form to potential subjects, allowing time for questions and discussion, and then assessing comprehension about the study via the teach-back method. Patients are also able to read the form independently if they wished. If comprehension questions during teach-back are not answered correctly, repeated education and reassessment of comprehension are continued until complete comprehension is achieved. If subjects cannot pass the teach-back process and comprehension assessment after three attempts, the patient is deemed ineligible for the study.

#### Electronic or written consent for clinicians to be audio-recorded

We obtain either an e-consent or written consent for clinicians to be audio-recorded during primary care visits, to answer one demographic question about their race/ethnicity, and to allow us to contact them in the future if we have further questions about their enrolled patients. The e-consent is emailed to clinicians on secure email servers. If clinicians cannot be reached by email, we obtain written consent in-person.

#### Verbal and written consent for surrogates

Some patients’ surrogate decision-makers may live outside of the area (in a different state), or cannot come to the VA for in-person informed consent. Initially, a waiver of signed consent for surrogates was not approved. At this time, a signed written consent form is required prior to any surrogate interviews. To accomplish this, we mail a consent form to the surrogate, review the consent form and conduct consent verification over the phone. The surrogate is then given a stamped and addressed envelope to return the signed consent form. After consent is obtained, we can then call and schedule a phone or in-person interview. This is time consuming and can result in several (up to 10) attempts to contact the surrogate. Therefore, we now obtain a written consent waiver for surrogates. After calling a potential surrogate participant, if they answer all questions on the consent verification accurately within three tries, the surrogate can provide verbal consent over the phone, and we can continue with the study interview. If the surrogate is available in person, then a written informed consent form is reviewed and signed. All surrogates are still required to complete and return a signed HIPAA authorization by mail for their interview information to be used in the study.

### Intervention and control conditions

After the baseline interview, participants in the PREPARE arm review the PREPARE website (www.prepareforyourcare.org) in the research offices. Research staff are available to answer questions, but participants are asked to go through the PREPARE website (Steps 1 through 5) on their own and in its entirety. As participants progress through the website, PREPARE asks questions about overall life values and goals for medical care. At the end of the program, the participant is asked to make an action plan to do one ACP task in the next weeks to months. At the end of the interview, research staff print out the action plan and a summary of the Veteran’s medical wishes and compile this into a folder that also contains the PREPARE pamphlet, booklet, DVD, and the log-in code to the website. The PREPARE DVD contains the same information found on the website, but without interactive or tailored functionality. PREPARE arm participants are also given an easy-to-read advance directive to review and take home (http://www.iha4health.org/our-services/advance-directive/) [13]. Participants in the control arm are only given the advance directive, are asked to review it for at least 10 minutes and to take it home. The control arm is not given any information about the PREPARE materials. One to 3 days before the Veteran’s next scheduled primary care appointment, research staff call the PREPARE arm participants to remind them to bring in their action plan, summary of wishes and advance directive to their doctor’s appointment. For the control arm, research staff members only remind Veterans about their upcoming primary care appointment.

### Randomization procedures

We block randomize Veterans based on limited health literacy, as determined by a validated question concerning confidence with medical forms [30], and non-white race/ethnicity to ensure these variables are equally distributed between randomization groups. A statistician not involved in recruitment or data collection used a computer-based random number generator to create a randomization scheme within four groups (high health literacy and white race/ethnicity, high health literacy and non-white race/ethnicity, low health literacy and white race/ethnicity, and low health literacy and non-white race/ethnicity) in random block sizes of 4, 6, and 8. This randomization scheme was imported into the Research Electronic Data Capture (REDCap) software by the statistician. Only the research personnel who conducted the baseline interview use this software prior to the baseline interview to determine which study arm the Veteran is assigned to and which ACP interventions (PREPARE versus control) to give the enrolled participant. Randomization information is kept separate from other research or identifying Veteran data and is only associated with a unique Veteran identification number.

### Blinding

Participants are blinded to group assignment. They are told that the purpose of the study is to help patients make difficult medical decisions, and that each participant would review one of two guides. This blinding is enhanced by each group obtaining some form of ACP materials, such as the easy-to-read advance directive. To ensure blinding of research staff for all follow-up outcome assessments, staff who had completed the baseline interview and randomization for a given participant will not conduct any follow-up interviews with that participant.

### Intervention fidelity

A study protocol manual was created for research staff, and several training sessions have been and will continue to be conducted until the end of the study. In addition, we have created standardized study scripts for recruitment and interviewing and have created training videos to standardize the study procedures. Checklists for all follow-up assessments have also been created. All staff being trained need to learn each item on the checklist, shadow other study staff, and be evaluated by their supervisors for each checklist item. Study staff cannot conduct PREPARE study tasks independently until they can demonstrate mastery of each checklist item. After study staff members are considered independent to conduct study tasks and interviews, a 10 % random sample of interviews will be observed to ensure fidelity to study procedures. Data collection initially began with paper surveys that were entered into a REDCap survey capture database. At least 50 % of the data entry was reviewed by an independent staff member to ensure data were entered correctly. Part way through our study, we switched to live data capture through REDCap. This allows us to streamline our data entry procedures and reduce the use of printed paper surveys. To reduce missing data, automatic prompts have been created within the REDCap program that will not allow study staff to progress if a question is left blank. Concerns about question wording or response option interpretation are reviewed at regular team meetings.

### Ethics

This study has been approved by the University of California, San Francisco (UCSF) and the San Francisco VA Medical Center institutional review boards (UCSF IRB reference # 10-00098) and is registered at ClinicalTrials.gov (NCT01550731). This study is funded by the United States Veterans Administration, Health Services Research and Development.

### Measures and data collection

A range of measures are collected to capture the full process of ACP and whether the PREPARE intervention has any effect on ACP, medical decision-making, patient activation, clinician responsiveness, and doctor-patient communication. Table 2 lists all study measures assessed at multiple time points. Details of the main outcome measures are described below.

**Table 2:** Constructs and measures for evaluating the efficacy of the PREPARE Study

Construct	Measure	# items	Reliability/validity	Screener	Baseline	1 week	3 month	6 month
	Eligibility screening variables							
Cognitive impairment	Short Portable Mental Status Questionnaire (SPMSQ)	7	Sensitivity 86.2 %, specificity 99.0 % [43]	X				
	0 to 2 = eligible							
	3 to 7 moderate impairment (go on to the Mini Cog three-item recall)							
	≥8 severe impairment = ineligible							
Cognitive impairment (participants scoring 3 to 7 errors on the SPMSQ)	Mini Cog (three-item recall as needed, if SPMSQ screen + for cognitive impairment)	3	Sensitivity 76 %, specificity 89 % [44]	X				
	If recall ≥ two words = eligible							
Vision	Ability to see words on a newspaper [42]	1						
	Moderator variables							
Demographic information	Age, gender, race/ethnicity [45], income, marital status, and education			X	X			
Health literacy screen	“How comfortable are you filling out medical forms by yourself?”	1	AUROC 0.80 (95 % CI = 0.67.0.93) for inadequate health literacy [46]	X				
	“Qué tan seguro (a) se siente al llenar formas usted solo (a)”							
Health literacy assessment	Short form Test of Functional Health Literacy in Adults s-TOFHLA, scores 0 to 36) [47] Continuous & dichotomized to limited = 0 to 22 and adequate = 23 to 36	36	Cronbach’s α = .97		X			
			Correlation coefficient w/ other literacy tests > 0.80 [47]					
United States acculturation	Based on Acculturation scale (USAS) “How many years have you lived in the U.S.?”	1	Cronbach’s α = .98		X			
			Associated w/ desire to know prognosis [48]					
Finances	“In general, how do your finances usually work out at the end of the month?”	1	Associated with functional impairment and comorbidity [49]		X			
Socioeconomic status and social standing	Social standing ladder (that is, place an “x” where you think you stand relative to other people in society)	1	Associated with functional decline [50]		X			
Functional status	Activities of Daily Living (ADL) and Instrumental Activities of Daily Living (IADL)	15	Morbidity/mortality correlation [51, 52]		X			
Self-rated health status	In general how would you rate your health? (5-pt Likert)	1	Correlation with global health, spearman’s rho = -63, and mortality [53]	X				
Self-rated quality of life	In general, how would you rate your overall quality of life in the past week (5-pt Likert)	1	Test-retest coefficient = 0.81 [54]	X				
Comorbid illness	Determined by ICD-9 codes (chart)	0	Mortality c-stat: [23]		X			
	Charlson comorbidity score [24]		Charlson = 0.704					
	Elixhauser comorbidity score [55]		Elixhauser = 0.793					
Social support	Modified Medical Outcomes Study Social Support (mMOS-SS)	11	Cronbach’s α = 0.88-.93 [56]		X			
Religion/spirituality	Self-reported extent of how spiritual/religious (5-pt Likert) and role play in decision-making.	4	Spirituality associated with quality of life. Religiosity associated with wanting all measures to extend life [57]		X			
Prior ACP experience	Prior ACP experiences (for example, Ever had to make life threatening medical decisions?”) [12]	5			X			
Major life changes	For example, “In the past 6 months, have you or someone close to you been faced with a serious medical problem or diagnosis?”	4						X
	Mediator variables^a^ (also measured as Outcome Variables)							
Baseline knowledge	Knowledge subscales of the ACP Engagement Survey.	6	Cronbach’s α = 0.84 (0.76-0.90), ICC = 0.70 (0.50-0.82) [15]		X			
Baseline self-efficacy	Self-efficacy subscales of the ACP Engagement Survey.	6	Cronbach’s α = 0.83 (0.75-0.89), ICC = 0.60 (0.41-0.76) [15]		X			
Baseline readiness	Readiness subscales of the ACP Engagement Survey.	10	Cronbach’s α = 0.92 (0.88-0.95), ICC = 0.60 (0.53-0.81) [15]		X			
Baseline barriers	Checkbox of 13 common barriers	13	Associated with ACP [25]		X			
Baseline attitudes	Processes of Change for ACP [16]	34	Responsive to an ACP intervention [15]		X			
Desired role in decision-making	Control Preference Scale (CPS) [58]	2	Correlation between preferred and actual role in decision-making [59–61]		X			X
	Primary Outcome Variables							
Full process of ACP	ACP Engagement Survey: Process Measures of knowledge, contemplation, self-efficacy, readiness	116	Process Measures: Cronbach’s α = 0.94 (0.91-0.96), ICC = 0.70 (0.54-0.82) [15]		X	X	X	X
	Action Measures: completion of advance directives, discussions		Action Measures: ICC = 0.87 (0.79-0.92) [15]					
	Secondary outcome variables							
Communication quality	Modified CAHPS (that is, did this provider explain things in a way that was easy to understand?)	14	Comparative Fit Index = 0.98, Tucker Lewis Index = 0.98		X	X		
			Internal consistency: 0.58 to 0.92. ≥ 0.70 for four of eight constructs [62]					
Satisfaction with communication	For example, “How satisfied are you that you could share your most important concerns with X/that X understood what was most important to you?)	8			X	X	X	X
Satisfaction with care	Care Consistent with Goals: Comparison of 10-point ratings about aggressiveness of care desired and care currently receiving.	4			X			X
Barriers to ACP	Checkbox of 13 common barriers (for example, thinking about the topic makes me nervous or sad; I am too healthy; I am too busy; my family or doctor is too busy; I prefer to leave my health in God’s hands; I don’t want to burden my family and friends; I want to leave the choice to my friends and family; I want to leave the choice to my doctors; and an open-category response for “other.”)	13	Associated with ACP [25]		X			X
Attitudes about ACP	Processes of change for ACP [16]	34	Responsive to ACP intervention [15]		X			
Desired role in decision-making	Control Preference Scale (CPS) [58]	2	Correlation between preferred and actual role in decision-making [59–61]		X			X
Satisfaction with decision making	Decisional Conflict Scale	20	test-retest coefficient = 0.81			X	X	X
			α coefficient: 0.78-0.92 for total scale. 0.58-0.92 for subscales [31]					
Depression and anxiety	Patient Health Questionnaire (PHQ-4)	4	Cronbach’s α = 0.78 [63]		X	X	X	X
Surrogate reports of patient engagement in ACP and other surrogate items	Modified from the ACP Engagement Survey [22], (for example, “Did [Veteran] ask you to be their surrogate decision maker, talk to you about leeway, talk to you about their values, tell other family or friends about their wishes, ask clinicians questions or have you ask clinicians questions?”	45						X
	Prior ACP	6						
	Care consistent with goals	4						
	Decisional Conflict	19						
	Implementation	13						
Implementation: acceptability	Acceptability and Usability		1 factor explained 81-85 % of variance/scale. Kuder-Richardson >0.75 [12]		X			
	(a) Ease of use and understanding	8						
	(b) Usefulness in decisions & discussions	6						
	(c) Attitudes about norms or expectations	6						
	for example, “Did you try to fill out the advance directive we gave you?” “Did you give it to a medical provider, social worker, or case manager?” If they respond no, “Why do you think you did not turn it in?” “What can we do to get other people to look over these materials?” “What would motivate them?” “What suggestions do you have to make these materials better?”							
Implementation: feasibility	Feasibility (Control) (for example, when and where to review ACP materials)	7						X
	Feasibility (PREPARE only) (for example, when and where to review ACP materials, and which PREPARE materials did you use and would recommend)	34						
	“Do you remember what your action plan was?”							
	“Did you complete your action plan?”							
	If no to completing an action plan, “Why do you think you have not completed your action plan?”							
	“After the first study visit, did you look at the (action plan, summary of your wishes, the PREPARE website, pamphlet, Booklet/or DVD) again?							
	If no, “Why do you think you didn’t you look at it?”							
	Satisfaction questions include “Which of the PREPARE materials was the most helpful?”; “Which would you use again?” “Which did you share with your decision maker, friends, or family?” “When is the best time to see the PREPARE materials?” “Where do you think most people would prefer to review the PREPARE materials (home, clinic, or public space)?							

^a^Whereas the mediator variables, measured at baseline, may explain how or why a particular effect or relationship occurs, these variables may also be affected by the intervention and are therefore also measured as outcome variables

#### Measures related to advance directive and ACP documentation

Standard ACP is measured by the completion of advance directives in the VA electronic medical record. We will assess baseline advance directive completion rates and the date the advance directive was signed as well as ACP discussions. At 6 months (date of the last follow-up interview), we reassess the advance directive completion rate and the date it was signed to determine length of time from study enrollment to documentation. An advance directive for the purposes of this study includes the VA or easy-to-read advance directive, a living will, a durable power of attorney for health care document (DPOAHC), a Physicians Orders of Life Sustaining Treatment form, or other documentation of the patient’s wishes for medical care (code status, such as full code or do not resuscitate or do not intubate orders by a physician).

#### Measures related to ACP engagement including behavior change process measures and ACP actions

Full ACP engagement is measured by Veteran self-report at baseline, 1 week, 3 months, and 6 months. Based on Social Cognitive Theory and Behavioral Change Theory [18, 19], we included measures to capture theoretical constructs related to contemplation of behavior, planning or intention to act on the behavior, and the behavior itself. If only the action behavior is measured, such as completion of an advance directive, clinically significant movement along the behavior change pathway will be missed. Thus, it is important to ascertain ACP process measures of behavior change (knowledge, contemplation, self-efficacy, and readiness) for several ACP actions in addition to whether they have completed an ACP behavior (Action Measures). These actions include 1) identifying of a surrogate, 2) identifying values and goals, 3) choosing the level of leeway or flexibility in decision-making for the surrogate, 4) and communicating this information with clinicians and surrogate decision makers, and 5) documenting one’s wishes [22]. As Veterans may have had varying degrees of experience with ACP, we assess baseline engagement in ACP within the past 5 years (standardized based on pilot data). We also ask about engagement in ACP since the randomization date, for example, “Within the past one week, 3 or 6 months”, depending upon the follow-up interval.

#### Validity and reliability of the ACP Engagement Survey

In a prior study, we established the validity and reliability of the ACP engagement survey in 50 older adults, aged 60 years or older with two or more chronic or serious illnesses (32 % female, 42 % non-white). Internal consistency of the survey was high with a Cronbach’s alpha of 0.94 for Process Measures. Seven-day test-retest reliability was also high with interclass correlations of 0.70 for Process Measures and 0.87 for Action Measures. We also tested discriminant validity by comparing process and action scores for older adults to healthy young adults and found statistically significant differences [22]. Furthermore, this survey has been used in pilot testing and has been shown to be able to detect ACP behavior change in response to the PREPARE website [15].

#### Measures related to engagement and satisfaction in decision-making

Engagement in decision-making is measured with two items (Table 2, desired role in decision-making). Satisfaction with decision-making is assessed with the Decisional Conflict scale for those who report a decision was made [31]. The Decisional Conflict scale consists of three subscales assessing decisional uncertainty, factors contributing to uncertainty, and decisional effectiveness.

#### Measures related to surrogate decision maker reports of Veteran engagement

Because the preparation guide is highly focused on communication with one’s surrogate, it is important to measure whether Veterans engage in preparation behaviors with his/her potential surrogate for important corroborating information. We ask yes/no questions for all 5 preparation domains (Table 2, surrogate reports of patient engagement in ACP and other surrogate items). Surrogates are also asked about their knowledge of the Veteran’s wishes and confidence in making decisions on the Veteran’s behalf on a 5-point Likert scale with response options from “not at all” to “extremely”.

#### Measures related to acceptability and usability of PREPARE versus control

Acceptability and usability of PREPARE compared to an advance directive alone are measured with validated scales from our prior work according to the following: (a) ease of use and understanding (8-item scale), (b) personal usefulness in treatment decisions and discussions (6-item scale), and (c) attitudes about norms or expectations (6-item scale) [12]. We also assess how comfortable the Veterans are in completing the forms and the website, how helpful they find the materials, and how likely they are to recommend the materials to others [32]. These outcomes are important to measure to ensure PREPARE will be used in clinical practice and in the community.

#### Measures related to Veteran activation within primary care encounters

Veteran activation within the clinical encounter is assessed by audio-recording primary care visits using Dr. Richard Street’s communication coding system. This system defines activation as the degree to which patients ask questions, express concerns, offer opinions, state preferences, introduce new topics, or make decisions [33]. These behaviors are considered active because they have been shown to influence clinician’s behavior and treatment decisions [19]. We are interested both in activation in general throughout the consultation and in any discussions of ACP [34]. Therefore, we will assess overall activation and ACP-specific activation. The number of activation utterances will be assessed by independent, blinded coders and will be included in a composite measure. Utterances will also be coded as “self-initiated” or “prompted”, depending on whether the activation was solicited or encouraged by the physician. An increase of one patient activation utterance is considered clinically relevant. The codes of patient activation will be transformed into quantitative data and stored in a relational database that can be exported for quantitative analysis. To protect against rater drift and decay, continuous monitoring of inter-rater reliability will occur throughout the entire coding period by double-coding 20 % of the conversations. The coders will resolve any coding disagreements by conference; the entire team will resolve persistent disagreements.

#### Measures related to clinician responsiveness within primary care encounters

Clinician’s responsiveness within the clinical encounter will be assessed by rating “informativeness”, participatory decision-making, and partnership building. Overall informativeness will be rated on a 5-point Likert scale by two independent raters from recordings using Dr. Street’s previously validated measure based on whether the clinician fully discusses what is causing the patient’s problem, the clinician explains everything to the patient, the clinician is very informative about the patient’s health, and the clinician’s recommendations are clear and easy to understand (score 4 to 20) [34, 35]. ACP-specific informativeness is adapted from this measure and uses the following three items: the clinician thoroughly explained everything about the ACP topic discussed, the clinician was very informative about ACP, and the clinician’s explanations about ACP are very clear (score 3 to 15). Clinician’s participatory decision-making/partnership building is adapted from Kaplan by Dr. Street [34, 35]. For this measure, independent raters will decide on a 1 to 10 scale (1 = not at all and 10 = a great deal) whether the clinician involved the Veteran in the decisions, gave the Veteran a sense of control over medical care, and asked the Veteran to take some responsibility for ACP or medical care (scores 3 to 30).

#### Measures related to barriers and facilitators of ACP and PREPARE dissemination

Perceived barriers to engagement in ACP behaviors are assessed with a 10-item survey of the common barriers defined in our prior work and other studies [25].

#### Input about implementation

Input about implementation is assessed at 6 months by asking Veterans in the PREPARE arm questions about the acceptability and usability of PREPARE (for example, when and where to review ACP materials, and which PREPARE materials did you use and would recommend). For Veterans in the control arm, we ask about implementation of the ACP materials (for example, when and where to review ACP materials). For Veterans in the PREPARE arm, similar questions are asked of their surrogate decision-makers about the Veterans’ use of the PREPARE materials and the surrogates’ opinions.

## Outcomes and Sample Size Calculations

### Primary outcome

The primary outcome is advance directive documentation in the medical record. This outcome was selected based on prior studies that have shown that advance directive interventions can increase documentation. A meta-analysis demonstrated a pooled effect size of 0.50 (95 % CI; 0.17-0.83) suggesting a positive effect associated with advance directive interventions [36]. One RCT of an ACP workbook in older Veterans also demonstrated an increase in living wills documented from 23 % in controls to 48 % in the intervention group (*P* < .001); and ACP notes from 24 % to 47 % (*P* < .001) [37]. Consistent with results from prior studies, our pilot study showed that a literacy appropriate advance directive doubled completion rates from baseline [12]. From SFVAMC administrative data, 11 % of the potential study sample had an advance directive in their medical record. We assume that after being included in the study, the control group may have an advance directive documentation rate of 15 %. If we assume doubling of the percent of ACP discussion notes between groups (what we consider clinically significant), a sample size of 350, or 175 in each arm, will afford us 92 % power with a two-tailed alpha of 0.05 to detect an elevation in rate of advance directive documentation from 15 % in controls to 30 % in the PREPARE group and 80 % power to detect a difference of 15 % in controls versus 27 % in the PREPARE group. After accounting for an estimated 15 % attrition rate, including death at 6 months (based on prior work) [12], our recruitment target is 410 Veterans or 205 in each arm.

### Secondary outcomes

The secondary outcomes of this study include a comprehensive range of five specific ACP behaviors: 1) identification of a surrogate, 2) identification of values and goals, 3) choosing the level of leeway or flexibility in decision-making for the surrogate, 4) communicating this information with clinicians and surrogate decision-makers, and 5) documenting one’s wishes. We hypothesize that the scores from these ACP engagement items could be 50 % higher in the PREPARE group compared to the control group. Based on a pilot, the mean engagement scores for older outpatients were 30.7 points out of 57 ± SD of 10. Therefore, we expect a mean score as high as 40 points, a 10-point average difference between PREPARE and controls. However, even an increase of three points may represent a new action in any of the five domains. Using a two-tailed alpha of 0.05, 175 veterans in each randomization group will give us > 99.9 % power to detect a 10-point difference in engagement score, 96 % power to detect a four-point difference, and 80 % power to detect a three-point difference. A sample size of 350 will also allow us to assess each of the five domains of the preparation engagement survey separately as dichotomous variables, “action taken/no action taken”. If we assume a doubling of the percent of Veterans who “took action” within each of the five domains, using a two-tailed alpha of 0.05, we estimate that 175 veterans in each randomization group will give 92 % power to detect a doubling in the percent of Veterans who “took action” from 15 % (controls) to 30 % (PREPARE group) and 88 % power to detect a difference of 15 % versus 27 %. Taking into account an anticipated 15 % drop out as describe above, our anticipated 410 Veterans or 205 in each arm will provide us ample power to assess these outcomes.

The sample size of 350 Veterans will also allow adequate power to assess interactions based on potential moderating variables (race/ethnicity, literacy, gender) for our outcomes. We also anticipate very low intra-clinician correlations due to clustering or contamination; randomization is at the patient level not at the clinician level, and intra-clinician correlation values for patient attitudes and knowledge are typically quite low even in physician-level cluster randomized trials (for example, 0.015) [38]. Furthermore, as we have a potential pool of 115 clinicians, most clinicians will care for no more than one to three eligible Veterans and thus, there will be negligible loss of effective sample size accounting for clustering and contamination.

In exploratory analysis to corroborate Veteran reports of activation, we will attempt to recruit 144 surrogates (72 in each arm) anticipating that 20 % of Veterans will not have identified a surrogate based on pilot data, 15 % of Veterans will be lost to follow-up, and 30 % of surrogates will refuse. For patient activation within clinical encounters, we will attempt to audio-record 200 Veterans (100 in each group) to account for potential 25 % of enrolled Veteran and 25 % clinician refusal rates. For input about implementation, we anticipate recruiting 175 Veterans in the PREPARE arm (accounting for a 15 % drop-out at 6 months) and 72 of PREPARE arm Veteran’s surrogates.

#### Timeline

**Fig. 2:**
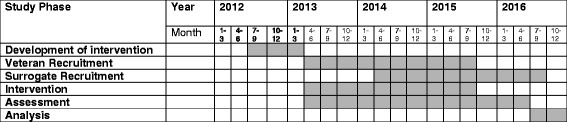
PREPARE Study Timeline

Figure 2 shows the timeline of the trial. We started recruitment in April 2013 and will end recruitment of Veterans in September 2015. We are currently undergoing recruitment of surrogate participants and expect to complete recruitment in September 2016.

### Data analysis

#### RCT efficacy analysis

Our primary analyses will compare advance directive completion rates and change in engagement in the five ACP behaviors (see Primary outcome section) using behavior change measure scores on a 5-point Likert scale (analyzed as average Likert scores and total scores of 57 to 285) and action measure scores using yes and no responses (scores 0 to 25) from baseline to 1 week, and 3, and 6 months between study arms. We first will use means, medians, and ranges to describe continuous variables, and we will use proportions to describe categorical and dichotomous variables. Baseline comparability of the two groups will then be assessed using t-tests for continuous variables and chi-square tests for proportions. To examine the outcomes between the two study arms longitudinally, we will use mixed effects linear, Poisson, or negative binomial regression for continuous outcome measures and mixed effects logistic regression for dichotomous measures. The mixed effects models will include a random effect for subjects and fixed effects for the primary modeling terms of time, study arm, and an interaction term of study arm and time. We will treat the time variable in three different ways: (i) our first model will encode the time variable as a dummy variable for baseline versus the post-intervention time points; (ii) we will next model time in a continuous linear fashion; (iii) we will lastly consider an arbitrary time course by treating time as a categorical factor variable. We will also adjust for the randomization blocking factors of health literacy (limited versus adequate) and race (white versus non-white) [39] and for any predictor variables that differ between study arms at baseline. We will also include random physician intercepts to account for nesting of patients within physicians. We will consider including random effects for the primary modeling terms of time, study arm, and their interaction. Modeling decisions (for example, which mean structure to use and which random effects to include) will be based on comparing values of the Akaike Information Criterion. Models will be fit using the xt routines in Stata. Mediation analyses will use Stata’s mediation package, which allows estimation of the average causal mediation effect using a potential outcomes framework with either continuous or binary outcomes and allows for sensitivity analyses with respect to violation of the sequential ignorability assumption [40]. Moderating variables will be tested with interaction terms.

#### Quantitative assessment of patient activation within clinical encounters

Differences in the number of patient activation utterances, ACP topics discussed, and length of time discussed will be compared between groups using mixed effects models as above with a random intercept for physician. Clinician race/ethnicity, and gender will be included as covariates as these variables are associated with doctor-patient communication [41]. We will also obtain the number of utterances that may signify clinician contamination (knowledge of study arm) to control for these findings.

## Discussion

This is the first study to test the efficacy of a new paradigm of ACP focused on preparing chronically ill older Veterans for communication and medical decision-making as operationalized in the PREPARE website. The development of the PREPARE was based on extensive published formative research in which the community, key stakeholders, and the target population were included in the development of the website. Designed to be easy-to-read and to include culturally competent material, PREPARE is also unique for its user-friendly features such as the use of video modeling of ACP behaviors, tailored and interactive content based on values and decision preferences, and the opportunity to create an action plan for change.

There have been a few logistic challenges to date. Although we attempt to schedule Veteran’s baseline appointment and randomization 1 to 3 weeks prior to their most proximate primary care appointment, this, at times, has been difficult to implement for various reasons. For example, some Veterans reschedule their baseline interview resulting in the intervention occurring within a few days before their clinic visit. Other Veterans or the clinic will cancel a primary care appointment after the baseline interview has occurred resulting in a time frame beyond the three-week ideal. We will adjust for these varying time frames from exposure to the intervention in our analyses. In addition, because the audio-recording occurs at the primary care visit and because several primary outcomes of the study would occur at the primary care visit, we decided to make the primary care visit the date from which all follow-up interviews would be calculated. Because follow-up of the 1-week, 3-month and 6-month interviews within defined time frames was proving challenging, we decided to allow a range of dates that were acceptable. For instance, if a primary care appointment is rescheduled is outside of the ideal 1-3 week window from the baseline interview and randomization, we will audio-record the primary care appointment up to 6 months after the baseline interview, but not after. For the one week follow-up interviews, the allowable Veteran interview range was changed from as early as 5 days from their primary care appointment to as late as 2 weeks prior to their 3-month follow-up; for the 3-month interview the range is from as early as 2.8 months from the primary care appointment to as late as 2 weeks before the 6-month interview; and for their 6-month interview, the ranges is from as early as 5.8 months from the primary care appointment to as late as 9 months from the appointment.

As described in the methods, surrogate recruitment was initially challenging without the ability to obtain verbal consent. However, this was approved by the IRB office part way through the study. Because surrogate contact takes a great deal of time, we have extended the surrogate recruitment date range to up to 6 months after the Veteran had completed their last 6-month follow-up visit. Even so, there have been several surrogates who were interested but never returned the consent materials, or who returned the consent materials but could not be scheduled before the 6-month cut off. We are hoping that verbal consent will make surrogate interviews easier to obtain.

In addition to testing PREPARE, we hope to be able to disseminate the website broadly. By assessing satisfaction with the PREPARE materials and suggestions for implementation and dissemination during the randomized trial phase, should the results be positive, we hope to move from the efficacy trial phase quickly to implementation and dissemination within the VA and the community.

If PREPARE proves efficacious in helping Veterans engage in ACP, communicate their wishes, better prepare themselves and their loved ones for complex medical decision-making, and document their wishes in the medical record, the PREPARE website could prove to be a scalable and effective intervention to improve the care of older Veterans and ensure Veteran’s wishes are honored. Because PREPARE can be used outside of the clinical environment, in future studies it may also save clinicians time and prove to be cost-effective.

### Trial status

This trial is in the active recruitment phase. While we have completed recruitment of 410 Veterans, we anticipate ending surrogate recruitment in September 2016.

## Abbreviations

ACP: advance care planning
SFVAMC: San Francisco Veterans Administration Medical Center
HIPAA: Health Insurance Portability and Accountability Act
RCT: randomized controlled trial
AD: advance directive
RedCAP: Research Electronic Data Capture
