# Erratum to: ‘The advance care planning PREPARE study among older Veterans with serious and chronic illness: study protocol for a randomized controlled trial’

**DOI:** 10.1186/s13063-016-1182-y

**Published:** 2016-01-20

**Authors:** Rebecca Sudore, Gem M. Le, Ryan McMahan, Mariko Feuz, Mary Katen, Deborah E. Barnes

**Affiliations:** Division of Geriatrics, Department of Medicine, University of California, San Francisco, 3333 California St. Suite 380, San Francisco, CA, 94143 USA; San Francisco Veterans Administration Medical Center, 4150 Clement Street, #151R, San Francisco, CA, 94121 USA; Division of General Internal Medicine, San Francisco General Hospital, Center for Vulnerable Populations, University of California, San Francisco, 2789 25th Street Suite 350, San Francisco, CA, 94110 USA; Department of Psychiatry, University of California, San Francisco, CA USA; Department of Epidemiology & Biostatistics, University of California, San Francisco, CA USA

Unfortunately, the original version of this article [1] contained an error. The author name was not spelt correctly. It was spelt as Ryan McMahon and should instead be Ryan McMahan.
